# DDPG-Based Throughput Optimization with AoI Constraint in Ambient Backscatter-Assisted Overlay CRN

**DOI:** 10.3390/s22093262

**Published:** 2022-04-24

**Authors:** Xueli Jia, Kechen Zheng, Kaikai Chi, Xiaoying Liu

**Affiliations:** School of Computer Science and Technology, Zhejiang University of Technology, Hangzhou 310023, China; 2111912158@zjut.edu.cn (X.J.); kkchi@zjut.edu.cn (K.C.); xiaoyingliu@zjut.edu.cn (X.L.)

**Keywords:** cognitive radio networks, ambient backscatter, age of information, DDPG

## Abstract

The combination of ambient backscatter (AB) communications (ABCs) and RF-powered cognitive radio networks (CRNs) deals with challenges of both energy supply and spectrum shortage, and improves the network performances. With the expansion of wireless networks, many applications raise requirements for both high-throughput and timely data. Driven by these facts, we study the long-term throughput optimization of the secondary network in the AB-assisted overlay CRN (ABO-CRN), ABCs, and CRNs with the age of information (AoI) constraint, which is a novel metric for measuring the freshness of data received by receivers. Due to the dynamic environment, complete knowledge of the environment could not be obtained. Then, the deep deterministic policy gradient (DDPG), a deep reinforcement learning (DRL) method that addresses decision issues in both continuous and discrete spaces, is deployed to address the throughput optimization. We consider the impacts of time and energy allocation on the reward when the AoI constraint can not be satisfied, and develop the corresponding reward functions. Furthermore, we analyze the impacts of the minimum throughput requirement and maximum allowable AoI on the throughput and AoI of the secondary networks in the ABO-CRN, ABCs, and CRNs. We compare the throughput optimization scheme under the AoI constraint with two baseline schemes (i.e., throughput-optimal (T-O) and AoI-optimal (A-O) baseline schemes), and the simulation results show that the throughput of the ABO-CRN is close to the optimal throughput of the T-O baseline scheme, and the AoI of the ABO-CRN is close to the optimal AoI of the A-O baseline scheme.

## 1. Introduction

Nowadays, the number of wireless devices has increased year by year, and the amount of spectrum demands has also increased [1]. However, the amount of spectrum is limited, and the majority of spectrum has been allocated to licensed users (primary users, PUs) as the licensed spectrum. PUs occupy the licensed spectrum at a certain time and places, and the licensed spectrum may not be occupied for a long time. In this case, the utilization ratio of the licensed spectrum is low [2]. In addition, due to the limited size, wireless devices can not carry large-capacity batteries, and frequent replacement of batteries is not allowed under certain circumstances, such as the inside of chimneys and the inside of bodies [3]. Therefore, issues of spectrum shortage and energy supply attract a large number of researchers. The RF-powered ambient backscatter (AB)-assisted cognitive radio networks (CRNs) (AB-CRNs) are introduced to improve the spectrum utilization ratio and alleviate the difficulty of energy supply. We introduce the RF-powered AB-CRNs from the following three aspects: the energy harvesting technology, the CRNs, and the AB communications (ABCs).

Energy harvesting technology, which allows wireless devices to scavenge energy from the environmental energy sources, is an efficient way to address the difficulty of energy supply [2,4]. The energy sources can be solar, wind, heat, and RF signals, etc. Some energy sources are unstable and conditionally available. Taking solar as an example, harvesting energy from solar depends on weather conditions, and can be used during the daytime. In addition, the devices powered by solar are bulky and costly [5], and most wireless devices can not carry huge solar panels. Differently, harvesting energy from RF signals has lots of advantages, i.e., stability, ubiquity, and controllability [6], and has been applied in a variety of wireless network fields. In [7], Yang et al. investigated the application of energy harvesting technology in wireless body area networks. Chi et al. [8] aimed to minimize the RF energy provision of time-division-multiple-access (TDMA) and non-orthogonal-multiple-access (NOMA) schemes in wireless powered communication network. In [9], Hoang et al. explored security and energy harvesting requirements in MIMO-OFDM networks. Authors in [10] and [11] applied energy harvesting technology in wireless sensor networks (WSN). Azarhava et al. [10] studied the energy harvesting WSN with TDMA scheme. Ghosh et al. [11] maximized the energy efficiency by proposing the upper confidence bound based algorithm.

Cognitive radio (CR) technology is introduced to deal with the issue of spectrum shortage. Secondary users (SUs), i.e., cognitive users, are allowed to access the licensed spectrum opportunistically without causing harmful influence to the PUs, which improves the spectrum utilization ratio [12]. SUs are able to execute various transmission modes in the CRNs, such as overlay mode and underlay mode. When secondary transmitters (STs) transmit data in overlay mode, they exclusively occupy the licensed spectrum when PUs are not using the licensed spectrum, and immediately leave the licensed spectrum when PUs begin to transmit. When STs transmit data in underlay mode, STs could access the licensed spectrum even when the licensed spectrum is used by PUs. However, the secondary transmission should not lower the transmission quality of PUs, hence the transmit power of STs can not exceed the power threshold that PUs tolerate [13]. RF-powered CRNs allow SUs to harvest energy from RF signals, and access the licensed spectrum following various transmission modes. As a result, RF-powered CRNs are able to alleviate both spectrum shortage and energy insufficiency, and have now been extensively studied. In [14], Zheng et al. divided a unit circular area into three concentric circle regions: overlay region, underlay region, and energy harvesting region, and examined spectrum-limited and energy-limited scenarios. In the cooperative CRN, Papadopoulos et al. [15] enhanced the overall throughput performance by two network coding algorithms. In [16], Rathee et al. considered security threats in the CRNs, and proposed a secure hand-off mechanism for the emulation attack of cognitive users.

Although the RF-powered CRNs have excellent performance in solving the problems of spectrum shortage and low utilization ratio, as well as energy supply difficulties, whether the transmission performance of the CRNs could be improved depends on the energy harvesting from the RF signals of primary users and the spectrum usage [17]. For example, if a ST harvests little energy, or its energy storage capacity is too small to store sufficient energy, the ST lacks energy to transmit data, or spends much time harvesting energy, degrading the throughput performance of the secondary network. Furthermore, if the PU occupies the licensed spectrum with high probability, the ST has few opportunities to transmit data. Driven by these facts, AB technology is proposed to alleviate difficulties of both spectrum shortage and energy insufficiency. AB mode transmission is performed by using ambient RF signals, such as TV signals, and it does not need extra spectrum. In addition, after receiving the RF signal, the devices with AB functionality map the data to a series of reflective states by adjusting the load impedance of the antenna, and then reflect the RF signals to the receiver according to the matching degree between the antenna impedance and the load impedance. The operation of reflection consumes little energy [18]. Ye et al. [19] investigated the ABCs with multiple channel links, and minimized the outage probability. Liu et al. [20] utilized coherent and non-coherent orthogonal space-time block codes to improve the backscatter efficiency in ABCs with multiple antennas. In [21], Madavani et al. studied a full-duplex ABC, and optimized the throughput of the minimum AB device and the overall throughput.

However, throughput of AB mode transmission is relatively low, especially when the primary RF signals are weak. Considering the characteristics of CR technology and AB technology, the combination of AB technology and RF-powered CRNs is potentially helpful for dealing with spectrum shortage and energy insufficiency. The RF-powered AB-CRNs are energy-saving and spectrum-saving [22]. As far as we know, Hoang et al. in [22] initially introduced the RF-powered AB-CRNs, and analyzed the throughput of the AB-assisted overlay CRN (ABO-CRN) and the AB-assisted underlay CRN (ABU-CRN). Extended from [22], Zhuang et al. [23] studied the RF-powered AB-CR-NOMA networks, where STs perform underlay mode transmission. In [24], Zhu et al. investigated the distributed resource allocation in AB-CRNs.

In [22,23], authors investigated popular metrics, such as throughput. Besides, the age of information (AoI), a novel metric that measures the freshness of data received at the receiver, has also been extensively studied in recent years [25]. Different from delay metric, AoI focuses on the data timeliness of the receiver, while delay metric focuses on that of the transmitter. Authors in [26,27,28] investigated the AoI minimization in the CRNs. In [26], Leng et al. utilized the partially observation Markov decision process to analyze the AoI performance in several cases. In [27], Gu et al. studied the AoI in overlay and underlay scenarios, and analyzed the effect of critical generation rate of the primary IoT on the secondary IoT. In [28], Wang et al. took the collision constraint into account to minimize the long-term average AoI. The AoI minimization challenge in ABCs is also a fascinating and important research topic. In [29], Abbas et al. focused on the minimization of AoI in the backscatter communications, and introduced several algorithms for the AoI minimization.

With the expansion of wireless networks, timely delivery is required [30], and in the scenarios, such as cyber-physical system, low-quality but timely data is useless [31]. However, the throughput optimization can not guarantee the freshness of data, and AoI optimization can not guarantee the quality of data. Liu et al. [32] investigated the AoI minimization under throughput requirements in the multi-path network. Kadota et al. [33] proposed a low-complexity scheduling algorithm for the AoI minimization with throughput constraints of the wireless network. Bhat  et al. [34] studied the throughput maximization under the AoI constraint in fading channels.

Obviously, compared with the optimization of a single frame, long-term optimization is more practical. In practice, the network environment is dynamic, and the channel quality, such as the channel gain, varies with the frame. The optimization of a single frame is limited by the channel quality, and affects that of the subsequent frames. The short-term optimization ignores the connection between the optimization of the current frame and that of subsequent frames, which degrades the performance of the network. However, the long-term optimization takes the aforementioned connection into account, and provides more practical decision to enhance the performance of the network. Taking the throughput optimization of a single frame by energy management as the example, the throughput increases with the consumed energy, hence consuming all the available energy of the frame is optimal for the short-term optimization. However, consuming all the available energy in the frame with poor channel quality leads to the lack of available energy in the frame with good channel quality. Therefore, the long-term optimization is more practical than the short-term optimization. Due to the dynamic and uncertain network parameters, the complete knowledge about the network could not be obtained in advance. Some traditional methods are incapable of addressing challenges with too many dynamic and unpredictable environmental parameters. Deep reinforcement learning (DRL) has been proved as an effective way to tackle the challenge [35,36]. When applying value-based DRLs, the action space for DRLs has to be discrete. If the discrete methods are improper for the scenario, vital information may be lost, or the action space dimension may be too large [37]. Different from value-based DRLs, DRLs based on the policy gradient are able to deal with the problems of continuous spaces, and have no need to discretize the action space. Policy gradient-based DRLs have been applied into the field of the wireless networks [38].

Taking the limitations summarized in Table 1 into account, we conclude the novelties and contributions as follows. Considering the problems of spectrum shortage and energy insufficiency, we focus on AB-CRNs, while a majority of researches in AB-CRNs evaluated the network performances such as throughput, energy consumption, delay, etc., and ignored the data freshness of the secondary receivers (SRs). Driven by the fact, we optimize the long-term throughput of the secondary network in the ABO-CRN with the AoI constraint, in order to guarantee the high throughput and data freshness of the secondary network. According to our knowledge, we are the first to study the optimization of both throughput and AoI in the research area of AB-CRNs. The main contributions are summarized as follows.

- In order to achieve the long-term throughput optimization of the secondary network with the AoI constraint, we utilize deep deterministic policy gradient (DDPG), a DRL based on the policy gradient, to find the optimal policy for jointly managing time and energy of STs. Considering the impacts of time and energy allocation on the reward when the AoI constraint can not be satisfied, we develop the corresponding reward functions with respect to the channel states.
- We analyze the minimum throughput requirement and the maximum allowable AoI for the throughput and AoI performances in the ABO-CRN, ABCs, and CRNs.
- We introduce throughput-optimal (T-O) and AoI-optimal (A-O) baseline schemes as comparisons for the throughput optimization with the AoI constraint. The simulation results show that the throughput of the ABO-CRN is close to the optimal throughput of the T-O baseline scheme, and the AoI of the ABO-CRN is close to the optimal AoI of the A-O baseline scheme.
- We evaluate the impacts of the minimum throughput requirement and maximum allowable AoI on the throughput and AoI performances of the secondary networks in the ABO-CRN, ABCs, and CRNs, and demonstrate that the ABO-CRN improves the throughput and AoI performances of the ABCs and CRNs.

The remainder of this paper is organized as follows. In Section 2, we introduce the network model and operations of STs in the ABO-CRN, ABCs, and CRNs. In Section 3, we introduce the problem formulation, such as throughput and AoI definitions. In Section 4, we utilize DDPG to find the optimal policy for jointly managing time and energy of STs. In Section 5, simulation results are shown. In Section 6, we conclude the paper.

## 2. System Model

In this section, we first depict the structures and channel models of the ABO-CRN, ABCs, and CRNs, and then introduce the network models of the ABO-CRN, ABCs, and CRNs, respectively.

### 2.1. Structures and Channel Models

The ABO-CRN, ABCs, and CRNs are composed of a primary network and a secondary network. The secondary network consists of a SR and n+1 STs, n∈{0,1,…}. In the primary network, the primary transmitter (PT) utilizes the licensed channel to transmit data. The probability that the PT occupies the channel in each frame, denoted by $P_{a}$, can be obtained through the long time observation. When the PT transmits data in frame t∈{1,2,…,K}, the channel state is active, denoted by $s_{t}^{a}=1$. When the PT does not transmit data in frame *t*, the channel state is inactive, denoted by $s_{t}^{a}=0$.

**Figure 1:**
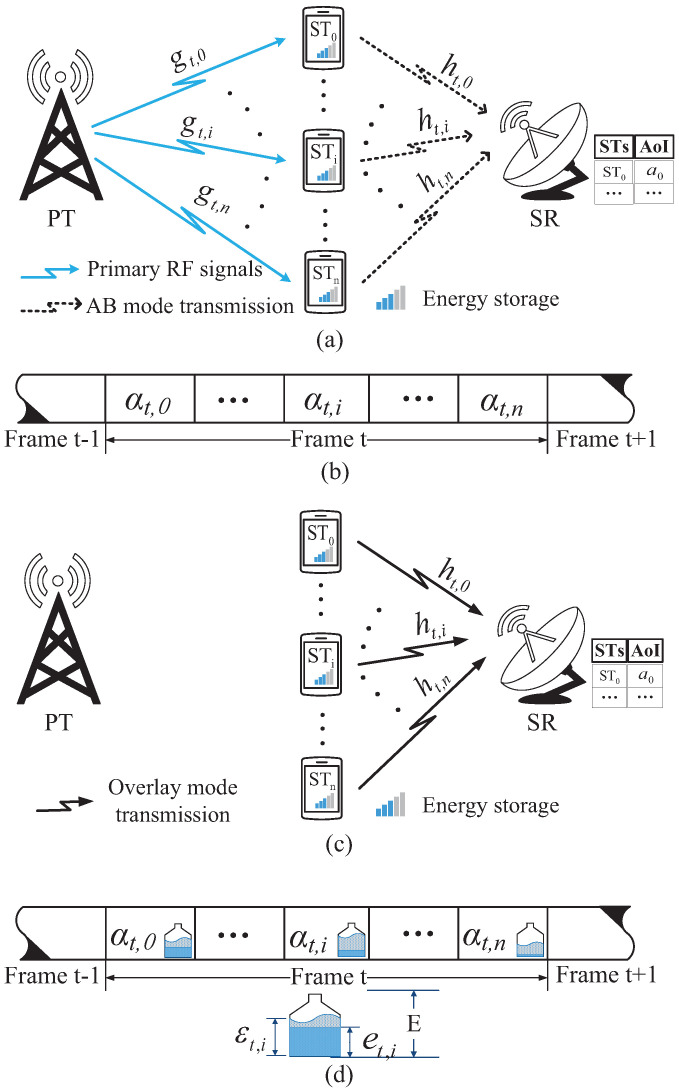
System model and frame structure of the AB-assisted overlay CRN: (**a**) depicts the system model when $s_{t}^{a}=1$, i.e., the channel is active. (**b**) depicts the time frame structure when $s_{t}^{a}=1$. (**c**) depicts the system model when $s_{t}^{a}=0$, i.e., the channel is inactive. (**d**) depicts the time frame structure when $s_{t}^{a}=0$.

In the secondary network, as the random distribution of the SUs in [39], the SR and STs are randomly placed within the coverage of the primary RF signals from the PT, as shown in the Figure 1a,c. Each ST is equipped with a single antenna and a rechargeable capacitor with finite capacity *E*. The SR is equipped with a single antenna and a wired energy source, hence there is no need to consider the energy supply of the SR. In order to measure the freshness of the data that the SR receives from STs, SR has capability to record the AoI of the data from each ST. Similar as [40], the SR plays the role of center controller to manage the time and energy for STs. At the very beginning of the frame, the SR senses the channel, and then provides the allocation of time and energy for STs.

**Figure 2:**
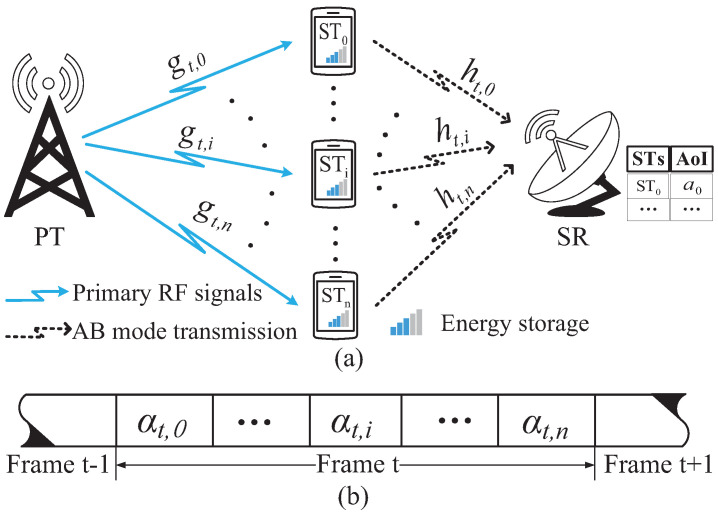
System model and frame structure of the ABCs: (**a**) depicts the system mode when $s_{t}^{a}=1$. (**b**) depicts the time frame structure when $s_{t}^{a}=1$.

**Figure 3:**
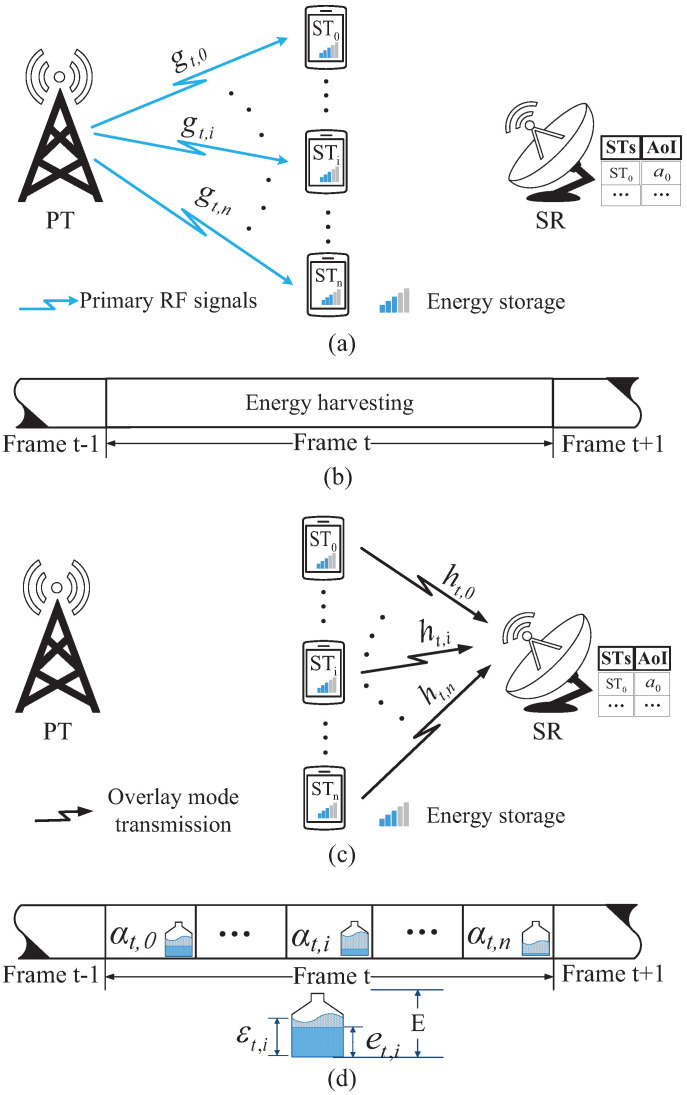
System model and frame structure of the CRNs: (**a**) depicts the system model when $s_{t}^{a}=1$. (**b**) depicts the time frame structure when $s_{t}^{a}=1$. (**c**) depicts the system model when $s_{t}^{a}=0$. (**d**) depicts the time frame structure when $s_{t}^{a}=0$.

In the considered scenario, frames with equal duration are successive. The frame duration is synchronized with the primary network, and without loss of generality, we normalize the frame duration as 1 [2]. As shown in Figure 1b,d, Figure 2b and Figure 3b,d, each frame consists of one or more slots. The duration of each slot is determined by the SR. We consider that the channel state remains unchanged in one frame, but varies in subsequent frames. When $s_{t}^{a}=1$, the SR and STs receive stable and continuous RF signals from the PT. In frame *t*, the channel gain between the PT and ST${}_{i}$, i=0,1,…,n, is denoted by $g_{t,i}$, and that between ST${}_{i}$ and the SR is denoted by $h_{t,i}$. The Rayleigh distribution [41] is used to formulate the channel gains that remain unchanged in one frame, and the channel noise is modeled as Additive White Gaussian Noise (AWGN) with variance $\delta^{2}$.

### 2.2. Network Models

#### 2.2.1. Network Model of ABO-CRN

The operations executed by the STs in the ABO-CRN depend on the value of $s_{t}^{a}$. When $s_{t}^{a}=1$, as shown in Figure 1a,b, STs execute AB mode transmission by TDMA scheme, and ST${}_{i}$ harvests energy when the other STs transmit data in AB mode. We consider the scenario where the energy consumption of AB mode transmission is negligible. Therefore, no dedicated slot for energy harvesting is required by STs. When $s_{t}^{a}=0$, as shown in Figure 1c,d, following the TDMA scheme, STs execute overlay mode transmission by consuming the energy stored in the rechargeable capacitor. With the aim to optimize the long-term average throughput with the AoI constraint, ST${}_{i}$ may not consume all the available energy $\varepsilon_{t,i}$ during frame *t*. The energy $e_{t,i}$ consumed by ST${}_{i}$, denoted by $e_{t,i}$, is determined by the SR.

**Figure 4:**
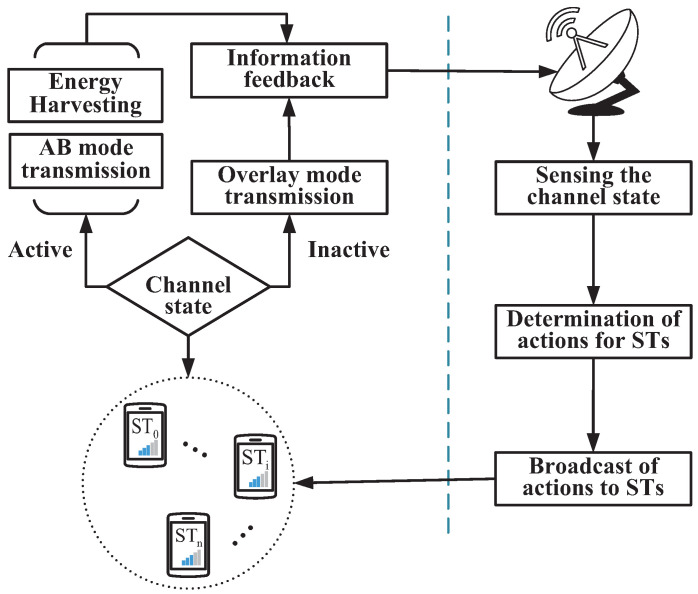
Flow chart of the AB-assisted overlay CRN.

We provide a flow chart of the ABO-CRN in Figure 4. The actions in Figure 4 are executed by STs according to $s_{t}^{a}$. The SR decides the time and energy allocation of STs according to the channel information and states of each ST. The channel information includes the channel state $s_{t}^{a}$ and channel gains. The states of each ST include the available energy and the AoI of the current frame. Note that, the feedback information in Figure 4 includes two parts. The first part is the new energy state in each ST, which is the available energy of the next frame. The second part is the received reward after STs execute the actions decided by the SR.

#### 2.2.2. Network Model of ABCs

The operations executed by the STs in the ABCs depend on the value of $s_{t}^{a}$. When $s_{t}^{a}=1$, as shown in Figure 2, STs take turns to execute AB mode transmission by TDMA scheme. When $s_{t}^{a}=0$, since the PT does not broadcast RF signals on the current channel, STs do not execute AB mode transmission.

#### 2.2.3. Network Model of CRNs

The operations executed by the STs in the CRNs depend on the value of $s_{t}^{a}$. When $s_{t}^{a}=1$, as shown in Figure 3a,b, STs harvest energy. When $s_{t}^{a}=0$, as shown in Figure 3c,d, following the TDMA scheme, STs execute overlay mode transmission by consuming the energy stored in the rechargeable capacitor. The energy $e_{t,i}$ consumed by ST${}_{i}$ is determined by the SR.

## 3. Formulation and Analysis of the Problem

For the readability, we provide a parameter list in Table 2 that summarizes the main parameters and meanings.

### 3.1. Throughput Definition

The total throughput $T_{t}$ of the secondary network in the ABO-CRN, ABCs, and CRNs in frame *t* can be expressed as

(1) $$T_{t}=\sum_{i=0}^{n}T_{t,i},$$

where $T_{t,i}$ denotes the throughput of ST${}_{i}$ in frame *t*. Due to the fact that the operations executed by STs depend on the channel state $s_{t}^{a}$, the calculation of $T_{t,i}$ depends on the value of $s_{t}^{a}$.

#### 3.1.1. Throughput Definition of ABO-CRN

In the ABO-CRN, $T_{t,i}$ is expressed as

(2) $$T_{t+1,i}=\left\{\begin{array}{cc} T_{t,i}^{A}, & \;\;s_{t}^{a}=1;\\ T_{t,i}^{O}, & \;\;s_{t}^{a}=0. \end{array}\right.$$

When $s_{t}^{a}=1$, according to the Shannon Theory, the throughput of ST${}_{i}$ by AB mode transmission, denoted by $T_{t,i}^{A}$, is expressed as

(3) $$T_{t,i}^{A}=\alpha_{t,i}W\log_{2}\left(1+\frac{\theta Pg_{t,i}h_{t,i}}{\delta^{2}}\right),$$

where $\alpha_{t,i}\in \left[0,1\right]$ denotes the duration of data transmission by ST${}_{i}$ through AB mode, *W* denotes the bandwidth, θ∈[0,1] denotes the backscatter reflection coefficient that depends on the electronic component factors, P denotes the transmit power of the PT, and $g_{t,i}$ denotes the channel gain from the PT to ST${}_{i}$, and $h_{t,i}$ denotes the channel gain from ST${}_{i}$ to the SR. In particular, $\theta Pg_{t,i}$ represents the transmit power of ST${}_{i}$ for AB mode transmission. ST${}_{i}$ harvests energy when the other STs transmit data in AB mode. The harvested energy of ST${}_{i}$, denoted by $e_{t,i}^{h}$, is calculated as

(4) $$e_{t,i}^{h}=\min\left\{\sum_{j=0,j\neq i}^{n}\alpha_{t,i}Pg_{t,i},\;E-\varepsilon_{t,i}\right\}.$$

After energy harvesting, the available energy in ST${}_{i}$ of frame t+1, denoted by $\varepsilon_{t+1,i}$, is updated as

(5) $$\varepsilon_{t+1,i}=\min\left\{\varepsilon_{t,i}+e_{t,i}^{h},E\right\}.$$

When $s_{t}^{a}=0$, according to the Shannon Theory, the throughput of ST${}_{i}$ by overlay mode transmission, denoted by $T_{t,i}^{O}$, is expressed as

(6) $$T_{t,i}^{O}=\alpha_{t,i}W\log_{2}\left(1+\frac{e_{t,i}h_{t,i}}{\alpha_{t,i}\delta^{2}}\right),$$

where $\alpha_{t,i}\in \left[0,1\right]$ denotes the duration of data transmission by ST${}_{i}$ through overlay mode, and $e_{t,i}\in \left[0,\varepsilon_{t,i}\right]$ denotes the energy consumed for overlay mode transmission in frame *t*. In particular, $e_{t,i}$ is determined by the SR, and $\varepsilon_{t+1,i}$ in ST${}_{i}$ of frame t+1 is updated as

(7) $$\varepsilon_{t+1,i}=\max\left\{\varepsilon_{t,i}-e_{t,i},\;0\right\}.$$

#### 3.1.2. Throughput Definition of ABCs

In the ABCs, the throughput of STs is achieved by AB mode transmission when the channel state is active. Therefore, when $s_{t}^{a}=0$, $T_{t,i}=0$ holds, and when $s_{t}^{a}=1$, according to the Shannon Theory, $T_{t,i}$ is expressed as

(8) $$T_{t,i}=T_{t,i}^{A}=\alpha_{t,i}W\log_{2}\left(1+\frac{\theta Pg_{t,i}h_{t,i}}{\delta^{2}}\right),$$

where $\alpha_{t,i}$, θ, P, $g_{t,i}$, and $h_{t,i}$ represent the same meaning as that in Equation (3). Since the energy consumption of AB mode transmission is negligible, the energy update is not considered in the ABCs.

#### 3.1.3. Throughput Definition of CRNs

In the CRNs, the throughput of STs is achieved by overlay mode transmission when the channel is inactive. When the channel is active, STs harvest energy from the RF signal of the PT. Therefore, when $s_{t}^{a}=1$, $T_{t,i}=0$ holds, and $\varepsilon_{t+1,i}$ in ST${}_{i}$ of frame t+1 is updated as

(9) $$\varepsilon_{t+1,i}=\min\left\{\varepsilon_{t,i}+e_{t,i}^{h},E\right\}.$$

When $s_{t}^{a}=0$, according to the Shannon Theory, $T_{t,i}$ is expressed as

(10) $$T_{t,i}=T_{t,i}^{O}=\alpha_{t,i}W\log_{2}\left(1+\frac{e_{t,i}h_{t,i}}{\alpha_{t,i}\delta^{2}}\right).$$

$\varepsilon_{t+1,i}$ in ST${}_{i}$ of frame t+1 is updated as that in Equation (7).

### 3.2. Definition of AoI

AoI is a novel metric to measure the freshness of data received by the receiver. In particular, AoI is used to track the time elapsed since the time point of the latest data generation to the time point that the latest data is successfully received by the receiver [33]. We utilize the linear scheme to calculate AoI of STs, where the AoI is updated as

(11) $$a_{t+1,i}=\left\{\begin{array}{cc} 1, & \;\;\lambda_{t,i}=1;\\ a_{t,i}+1, & \;\;\lambda_{t,i}=0, \end{array}\right.$$

where $a_{t,i}$ denotes the AoI of ST${}_{i}$ in frame *t*, $\lambda_{t,i}=1$ indicates that the latest data of ST${}_{i}$ is successfully received by the SR, and $\lambda_{t,i}=0$ indicates that the latest data of ST${}_{i}$ is not successfully received by the SR. With the aim to optimize the long-term average throughput of the secondary network with the AoI constraint, we set a minimum throughput requirement $T_{min}$ for every ST. Specifically, if the throughput of ST${}_{i}$ during frame *t* is no less than $T_{min}$, the latest transmitted data of ST${}_{i}$ is considered to be successfully received by the SR. Based on Equation (11) and the aforementioned analysis of $\lambda_{t,i}$, $\lambda_{t,i}$ is expressed as

(12) $$\lambda_{t,i}=\left\{\begin{array}{cc} 1, & \;\;T_{t,i}\geq T_{min};\\ 0, & \;\;\mathrm{else}. \end{array}\right.$$

By combining Equations (11) and (12), the update of AoI is calculated as

(13) $$a_{t+1,i}=\left\{\begin{array}{cc} 1, & \;\;T_{t,i}\geq T_{min};\\ a_{t,i}+1, & \;\;\mathrm{else}. \end{array}\right.$$

Obviously, when $s_{t}^{a}=0$ in the ABCs, STs achieve negligible throughput, hence the throughput of each ST can not exceed $T_{min}$, $a_{t+1,i}=a_{t,i}+1$ holds. When $s_{t}^{a}=1$ in the CRNs, the same conclusion holds.

### 3.3. Problem Formulation

The throughput optimization objective function of the ABO-CRN, ABCs, and CRNs is expressed as

(14a) $$\begin{array}{cc} \mathrm{Maximize}\; & \bar{T}=\lim_{K\to \infty }\frac{1}{K}E\left(\sum_{t=1}^{K}T_{t}\right) \end{array}$$

(14b) $$\begin{array}{cc} s.t.:\; & 0\leq \alpha_{t,i}\leq 1\;\mathrm{and}\;\sum_{i=0}^{n}\alpha_{t,i}\leq 1, \end{array}$$

(14c) $$\begin{array}{cc} & 0\leq e_{t,i}\leq \varepsilon_{t,i}, \end{array}$$

(14d) $$\begin{array}{cc} & \lim_{K\to \infty }\frac{1}{K(n+1)}E\left(\sum_{t=1}^{K}\sum_{i=0}^{n}a_{t,i}\right)\leq A_{max}, \end{array}$$

where $A_{max}$ denotes the maximum allowable AoI that the secondary network tolerates, and Equation (14d) indicates that the average accumulated AoI should be smaller than $A_{max}$. Since the energy consumed by AB mode transmission of STs is negligible, the SR in the ABCs does not need consider the constraint in Equation (14c).

### 3.4. Analysis of $T_{min}$ and $A_{max}$

In this subsection, we analyze $T_{min}$ and $A_{max}$ in the ABO-CRN, ABCs, and CRNs. The expectation of the long-term average throughput in the ABO-CRN, ABCs, and CRNs is expressed as

(15) $$E\left(\bar{T_{t,i}}\right)=\lim_{K\to \infty }\frac{1}{K(n+1)}E\left(\sum_{t=1}^{K}\sum_{i=0}^{n}T_{t,i}\right).$$

According to Equations (2), (8) and (10), we have

(16) $$E\left(\sum_{t=1}^{K}\sum_{i=0}^{n}T_{t,i}\right)=\left\{\begin{array}{cc} E\left(\sum_{t=1}^{K}\sum_{i=0}^{n}\left(P_{a}T_{t,i}^{A}+\left(1-P_{a}\right)T_{t,i}^{O}\right)\right), & \mathrm{ABO}\text{-}\mathrm{CRN};\\ E\left(\sum_{t=1}^{K}\sum_{i=0}^{n}P_{a}T_{t,i}^{A}\right), & \mathrm{ABCs};\\ E\left(\sum_{t=1}^{K}\sum_{i=0}^{n}\left(1-P_{a}\right)T_{t,i}^{O}\right), & \mathrm{CRNs}. \end{array}\right.$$

$T^{-}$ denotes the long-term average throughput of each ST whose average throughput is smaller than $T_{min}$, $T^{+}$ denotes that is no smaller than $T_{min}$, *a* denotes the long-term average AoI of each ST whose average throughput is smaller than $T_{min}$, and let *N* equal n+1.

**Lemma** **1.** *With $E\left(\bar{T_{t,i}}\right)\leq T_{min}$ holds, when $T^{-}$ is closer to $T_{min}$, the tolerable value interval of a is larger. When $a>A_{max}-1$, the AoI constraint can not be satisfied.*

**Proof.**  We assume there are *x* STs with $T^{-}$, and N−x STs with $T^{+}$. We have

(17) $$E\left(\bar{T_{t,i}}\right)=\frac{xT^{-}+\left(N-x\right)T^{+}}{N}=\frac{x\left(T^{-}-T^{+}\right)+NT^{+}}{N}.$$

Since $E\left(\bar{T_{t,i}}\leq T_{min}\right)$ holds, we have

(18) $$E\left(\bar{T_{t,i}}\right)-T_{min}=\frac{x\left(T^{-}-T^{+}\right)+NT^{+}}{N}-T_{min}\leq 0,\;\mathrm{and}\;x\geq \frac{N(T-T^{+})}{T^{-}-T^{+}}.$$

In order to satisfy the AoI constraint, Equation (14d) is updated to $\frac{x(a+1)+N-x}{N}$, and we have

(19) $$\frac{x(a+1)+N-x}{N}-A_{max}\leq 0,\;\mathrm{and}\;a\leq \frac{(A_{max}-1)N}{x}.$$

Bring $x=\frac{N(T-T^{+})}{T^{-}-T^{+}}$ into Equation (19), and we have

(20) $$a\leq \left(A_{max}-1\right)\frac{T^{-}-T^{+}}{T-T^{+}}.$$

Obviously, when $T^{-}$ is closer to $T_{min}$, the tolerable value interval of *a* is larger.

Since $T^{-}<T$ holds, the $\frac{T^{-}-T^{+}}{T-T^{+}}<1$ holds, hence *a* can not exceed $A_{max}-1$. Therefore, when $a>A_{max}-1$, the AoI constraint can not be satisfied. The proof is completed. □

**Lemma** **2.** *The lower bound of $A_{max}$ that makes STs satisfy the Equation (14d) decreases with n, and increases with the number of STs whose average throughput is smaller than $T_{min}$.*

**Proof.**  We assume *x* is the number of STs whose average throughput is smaller than $T_{min}$, *a* has been given. From Equation (19), we deduce

(21) $$A_{max}\geq \frac{ax}{n+1}-1.$$

With the larger value of *n*, the lower bound of $A_{max}$ that makes STs satisfy the Equation (14d) decreases. With the larger value of *x*, the lower bound for $A_{max}$ increases. The proof is completed. □

Then we compare the impacts of $T_{min}$ and $A_{max}$ on the ABO-CRN, ABCs, and CRNs. We discuss the impacts in some extremely cases, i.e., $P_{a}$, the probability of $s_{t}^{a}=1$, is relatively small or relatively large. In the ABCs, when $P_{a}$ is relatively small, there are few opportunities for AB mode transmission. In the CRNs, when $P_{a}$ is relatively large, there are few opportunities for overlay mode transmission. In these two cases, $E\left(\bar{T_{t,i}}\right)$ is small, and the lower bound of $T_{min}$ is low, and the tolerable value interval and $A_{max}$ is small. Different from the ABCs and CRNs, when $s_{t}^{a}=1$, the STs in the ABO-CRN execute AB mode transmission, and when $s_{t}^{a}=0$, STs execute overlay mode transmission. As described in Equation (16), $E\left(\bar{T_{t,i}}\right)$ of the ABO-CRN is higher than that of the ABCs and CRNs. Under the same conditions of $P_{a}$, $T_{min}$, and $A_{max}$, the ABO-CRN achieves higher throughput while satisfying the AoI constraint.

## 4. Policies of Time and Energy Management

As described in the introduction, long-term optimization is more practical than the optimization of a single frame. Maximizing the throughput of a single frame with the AoI constraint may not be desirable. As a result, we consider the long-term optimization of the throughput. However, since network environmental factors, such as the channel state and channel gains, are dynamic and uncertain, it is difficult for SUs to obtain complete knowledge about the network environmental factors in advance. DRL is an excellent way to tackle the challenge. For some DRLs, such as deep Q-learning network (DQN) that is based on the value-function policy, discrete spaces are necessary. If the discrete methods are not suitable for the scenario, it may lose important information, or lead to the high space dimension. Therefore, we utilize DDPG, which deals with problems of continuous spaces, to find the optimal policy of time and energy management for throughput optimization. We define the details about DDPG in the following subsections.

### 4.1. Definitions of Spaces and Rewards

The SR plays the role of agent that provides decisions for STs. According to Equations (2)–(14d), the state spaces, action space, and rewards are introduced as follows.

#### 4.1.1. State Space

The SR determines time and energy allocation of STs based on the states of STs and channel information of the current frame, including the available energy in STs, the AoI about STs, channel gains, and the channel state. Therefore, the state space contains information about energy states, AoI states, states of channel gains, and channel states. The energy-state space is represented by

(22) $$S_{E}=\left\{\left(\varepsilon_{t,0},\varepsilon_{t,1},\ldots ,\varepsilon_{t,n}\right);0\leq \varepsilon_{t,i}\leq E\right\}.$$

The AoI-state space is represented by

(23) $$S_{A}=\left\{\left(a_{t,0},a_{t,1},\ldots ,a_{t,n}\right)\right\},$$

where the average accumulated AoI satisfies Equation (14d). In order to reduce the dimension of the channel-gain-state space, we represent the channel gains as

(24) $$\begin{array}{cc} h_{t,i} & =10^{-3}\eta_{h,t}l_{i}^{\epsilon },\\ g_{t,i} & =10^{-3}\eta_{g,t}L_{i}^{\epsilon }, \end{array}$$

where $\eta_{h,t}$ denotes the path loss coefficient from the PT to STs, and $\eta_{g,t}$ denotes the path loss coefficient from STs to the SR, $l_{i}$ denotes the distance between the PT and ST${}_{i}$, and $L_{i}$ denotes the distance between the SR and ST${}_{i}$, and ϵ denotes the channel path fading exponent. Therefore, the channel-gain-state space is represented by

(25) $$S_{G}=\left\{\left(\eta_{h,t},\eta_{g,t}\right)\right\},$$

where $\eta_{h,t}$ and $\eta_{g,t}$ follow the Rayleigh distribution. The channel-state space is expressed as

(26) $$S_{C}=\left\{s_{t}^{a};s_{t}^{a}\in \left\{0,1\right\}\right\},$$

where $P_{a}$ represents the probability of $s_{t}^{a}=1$.

In summary, the state space of the ABO-CRN and of the CRNs when $s_{t}^{a}=0$ is expressed as

(27) $$S=S_{E}\times S_{A}\times S_{G}\times S_{C}.$$

The state space of the ABCs when $s_{t}^{a}=1$ is expressed as

(28) $$S=S_{A}\times S_{G}\times S_{C}.$$

Note that, when $s_{t}^{a}=0$, STs in the ABCs do not execute AB mode transmission. When $s_{t}^{a}=1$, STs in the CRNs only harvest energy. As a result, the SR does not need to determine actions for STs, hence we do not design state space for these two cases.

#### 4.1.2. Action Space

In the ABO-CRN, based on the state of the current frame, the SR determines the actions that STs execute in the current frame. When $s_{t}^{a}=1$, STs execute AB mode transmission, and ST${}_{i}$ harvests energy when the other STs transmit data in AB mode. When $s_{t}^{a}=0$, STs execute overlay mode transmission. We define the action space as

(29) $$A=\left\{\begin{array}{c} \left(\alpha_{t,0},\alpha_{t,1},\ldots ,\alpha_{t,n},e_{t,0},e_{t,1},\ldots ,e_{t,n}\right);\sum_{i=0}^{n}\alpha_{t,i}\leq 1,\;0\leq e_{t,i}\leq \varepsilon_{t,i}. \end{array}\right\}$$

**Figure 5:**
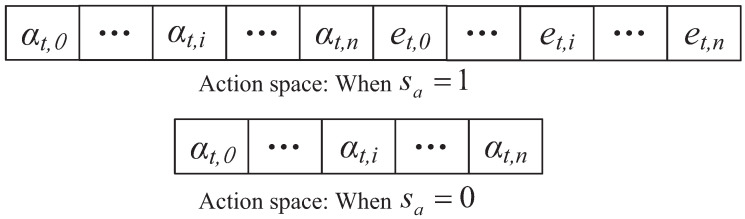
The action space diagram.

In particular, when $s_{t}^{a}=1$, the SR only utilizes the time $\left(\alpha_{t,0},\alpha_{t,1},\ldots ,\alpha_{t,n}\right)$ of *A* to address the time management for STs, which is shown as Figure 5.

When $s_{t}^{a}=1$, STs in the ABCs execute AB mode transmission. Since the energy consumption of AB mode transmission can be ignored, the SR in the ABCs focuses on the time allocation of STs. We define the action space of the ABCs as

(30) $$A=\left\{\left(\alpha_{t,0},\alpha_{t,1},\ldots ,\alpha_{t,n}\right);\sum_{i=0}^{n}\alpha_{t,i}\leq 1.\right\}$$

When $s_{t}^{a}=0$, STs in the CRNs execute overlay mode transmission. The action space of the CRNs is defined as Equation (29).

#### 4.1.3. Rewards

After the SR determines the action $x_{t}$ based on the state $s_{t}$ of frame *t*, an immediate reward $r_{t}\left(s_{t},x_{t}\right)$ is obtained, where $r_{t}\left(s_{t},x_{t}\right)$ represents the evaluation of choosing $x_{t}$ under $s_{t}$. With the aim to optimize the throughput of the secondary networks with the AoI constraint, $r_{t}\left(s_{t},x_{t}\right)$ is defined as

(31) $$r_{t}\left(s_{t},x_{t}\right)=\frac{1}{n+1}\left(\frac{T_{t}}{T_{min}}-\sum_{i=0}^{n}\rho_{t,i}\right),$$

where $\rho_{t,i}$ denotes the penalty that is related to the AoI of ST${}_{i}$. Since the actions vary with respect to the value of $s_{t}^{a}$, the values of $\rho_{t,i}$ vary with respect to the value of $s_{t}^{a}$. When $s_{t}^{a}=1$, we have

(32) $$\rho_{t,i}=\left\{\begin{array}{cc} a_{t,i}, & \;\;T_{t,i}\geq T_{min};\\ a_{t,i}^{2}, & \;\;\mathrm{else}. \end{array}\right.$$

When $s_{t}^{a}=0$, we have

(33) $$\rho_{t,i}=\left\{\begin{array}{cc} a_{t,i}, & \;\;T_{t,i}\geq T_{min};\\ a_{t,i}^{2}m\frac{e_{t,i}}{E}\alpha_{t,i}, & \;\;\mathrm{else}, \end{array}\right.$$

where *m* is a constant that is set according to $A_{max}$, and is used to ensure that $\rho_{t,i}$ is larger than $A_{max}$. $\frac{e_{t,i}}{E}\alpha_{t,i}$ indicates that the penalty increases with $e_{t,i}$ and $\alpha_{t,i}$ when $a_{t,i}$ is larger than $A_{max}$. The SR in the ABCs does not determine actions of the energy allocation for STs, hence we set $\frac{e_{t,i}}{E}$ in Equation (33) as $\frac{e_{t,i}}{E}=1$.

### 4.2. Time and Energy Management by DDPG

DDPG utilizes the architecture of the actor-critic algorithm and the scheme of DQN. Therefore, DDPG consists of two parts, actor and critic. The actor is used to output a deterministic action, and the critic is used to output an evaluation, which fits the Q-table. Both actor and critic consist of evaluated networks and target networks. The target networks make the training process more stable, and have the same structure with the evaluated networks. The evaluated network of the actor is named as the actor network, and that of the critic is named as the critic network. The target network of the actor is named as the actor target network, and that of the critic is named as the critic target network.

These networks are expressed as parametric functions. The actor network is expressed as a function mapping $s_{t}$ to $x_{t}$,

(34) $$x_{t}=\Pi \left(s_{t}|\omega \right),$$

where Π denotes the policy of time and energy management, and ω denotes the weights of neural network in the actor network. The critic network is expressed as an action-value function, which maps $s_{t}$ and $x_{t}$ to a *Q*-value,

(35) $$Q=Q(s_{t},x_{t}|\mu ),$$

where μ denotes the weights of the neural network in the critic network. Furthermore, the *Q*-value function is expressed as

(36) $$Q=E[r_{t}\left(s_{t},x_{t}\right)+\gamma \left[Q\left(s_{t+1},\Pi \left(s_{t+1}|\omega^{+}\right)|\mu^{+}\right)\right]],$$

where $\omega^{+}$ denotes the weights of the neural network in actor target network, $\mu^{+}$ denotes the weights of the neural network in critic target network, and γ∈[0,1] denotes the discounting factor, which represents the effect of the future action choices.

In order to weaken the dependence of DDPG on hyper-parameters, the batch normalization [42] is adopted for DDPG, i.e., each layer in the neural networks of DDPG is connected to a batch normalization layer, which makes the DDPG less sensitive to the initial parameters, and prevents the unstable training process resulted from the unstable data distributions of each layer in the neural networks. The batch normalization accelerates the converge of DDPG, and efficiently avoids the gradient vanishing. Furthermore, due to the different value ranges of each factor in states, we normalize the input state of DDPG so that each factor in the state has the same value range.

Algorithm 1 finds the optimal policy for the time and energy management by DDPG. The exploration noise $N_{t}^{e}$ in Algorithm 1 is used to fully explore the action space, in order to avoid being stuck in the local optimum policy. In the training process, the exploring noise decay factor κ restricts the exploration range. The weights $\omega^{+}$ and $\mu^{+}$ of the target networks are updated by the soft replacement that increases the stability of the evaluated networks.
**Algorithm 1:** Finding the optimal policy for the time and energy management by DDPG. 
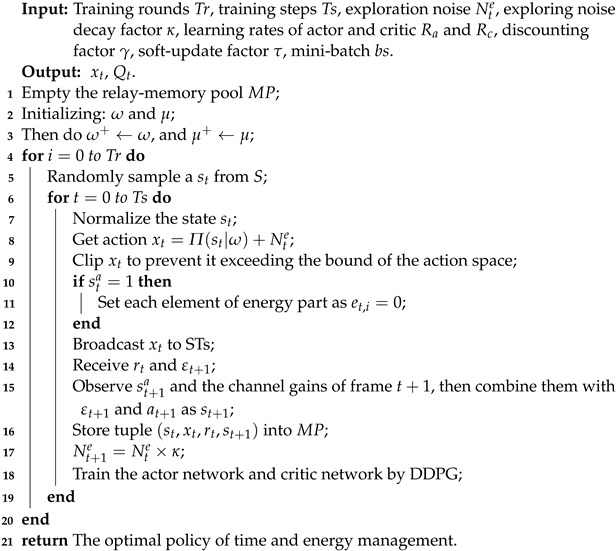


## 5. Simulation

In order to evaluate the performances of throughput and AoI, we compare the long-term average throughput T of the ABO-CRN with the AoI constraint with two baseline schemes, throughput-optimal (T-O) scheme and AoI-optimal (A-O) scheme. The T-O baseline scheme optimizes the throughput of the secondary network, and the A-O baseline scheme optimizes the AoI of STs. Furthermore, we compare the throughput and AoI performances among the ABO-CRN, ABCs, and CRNs to evaluate the impacts of $T_{min}$ and $A_{max}$ on the throughput and AoI performances. The simulation configuration is set as follows unless otherwise specified: The transmit power of PT P=17 kW, the bandwidth W=6 MHz, the AWGN $\delta^{2}=10^{-3}\;\mu W$, the energy capacity E=30μJ, and backscatter reflection efficiency θ=0.9.

**Figure 6:**
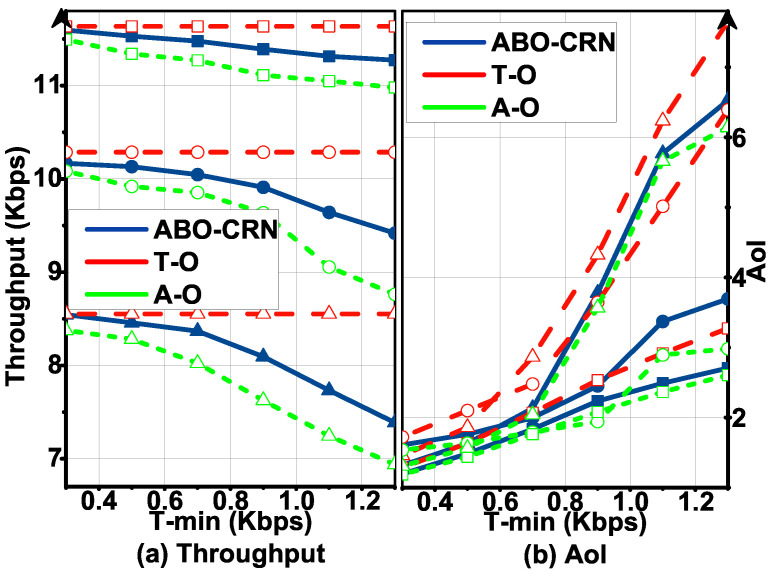
Throughput T and AoI versus $T_{min}$ under $P_{a}=$ 0.3 (circle), 0.6 (square), 0.9 (triangle) compared with that of T-O and A-O baseline schemes, and $A_{max}=5$: (**a**) describes the throughput performance. (**b**) describes the AoI performance.

Figure 6 plots T and AoI of the ABO-CRN, T-O baseline scheme, and A-O baseline scheme with the minimum throughput requirement $T_{min}$ under $P_{a}=$ 0.3, 0.6, 0.9. We observe from Figure 6a that T of the ABO-CRN decreases with $T_{min}$, and observe from Figure 6b that AoI of the ABO-CRN increases with $T_{min}$. For T-O baseline scheme, the throughput does not change with $T_{min}$, and the AoI increases faster than that of the ABO-CRN and A-O baseline scheme. The throughput of A-O baseline scheme decreases faster with $T_{min}$ than that of the ABO-CRN. The AoI of A-O baseline scheme increases with $T_{min}$, is close to that of the ABO-CRN, and is lower than that of T-O baseline scheme. The reasons can be explained as follows. When $T_{min}$ increases, each ST needs more throughput to reach the minimum throughput requirement. The SR in the ABO-CRN has to allocate more time and energy for the STs with high AoI and poor channel quality, and sacrifices the total throughput to satisfy the AoI constraint.

When $P_{a}=0.9$, we observe that T in Figure 6a decreases faster than that when $P_{a}=$ 0.3 and 0.6, and the corresponding AoI in Figure 6b increases faster. The reason is provided as follows. When $P_{a}=0.9$, due to the active channel state for the most time, STs only execute AB mode transmission for the most time. When $T_{min}$ is higher than the expected throughput achieved by STs through AB mode transmission, the AoI increases for the most frames, and the number of frames of the increased AoI becomes more with $T_{min}$. Therefore, the average AoI increases with $T_{min}$. The expected throughput achieved by STs through AB mode transmission and overlay mode transmission when $P_{a}=$ 0.3 and 0.6 is higher than that when $P_{a}=$ 0.9. Therefore, the average AoI when $P_{a}=$ 0.9 increases faster with $T_{min}$ than that when $P_{a}=$ 0.3 and 0.6. We also observe that when $T_{min}$ is small, the curves of the ABO-CRN and two baseline schemes are close. The reason is that, the throughput of three schemes meets the minimum throughput requirement, and the AoI of them satisfies the AoI constraint. In addition, Figure 6 shows that T of the ABO-CRN is closer to that of the T-O than that of A-O, and the AoI of the the ABO-CRN is closer to that of the A-O than that of T-O. It indicates that DDPG finds the optimal policy of time and energy management to optimize the throughput, and satisfies the AoI constraint.

**Figure 7:**
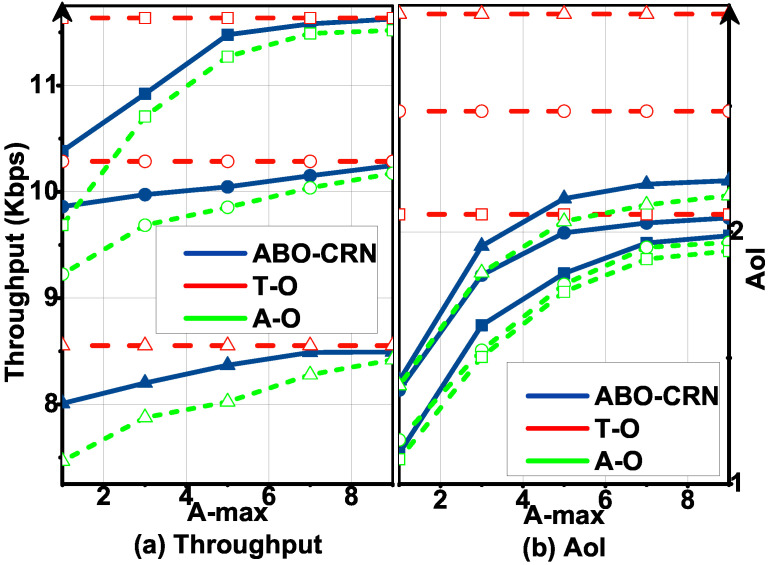
Throughput T and AoI versus $A_{max}$ under $P_{a}=$ 0.3 (circle), 0.6 (square), 0.9 (triangle) compared with that of T-O and A-O baseline schemes, and $T_{min}=700$ bps: (**a**) describes the throughput performance. (**b**) describes the AoI performance.

Figure 7 plots T and AoI of the ABO-CRN, T-O baseline scheme, and A-O baseline scheme with the maximum allowable AoI constraint $A_{max}$. We observe that both T and AoI increase with $A_{max}$, and when $A_{max}$ is large, the throughput of the ABO-CRN and that of two baseline schemes are close. The reason is provided as follows. When $A_{max}$ increases, the limitation of the AoI constraint on throughput becomes weak. The SR allocates more time and energy to the STs with high throughput, hence the throughput increases with $A_{max}$. With the increase of $A_{max}$, STs with high AoI (but not exceed $A_{max}$) and poor channel quality is allocated less time and energy, hence AoI of these STs increases, and the average AoI of STs increases. When $A_{max}$ is large, all the three schemes satisfy the AoI constraint.

**Figure 8:**
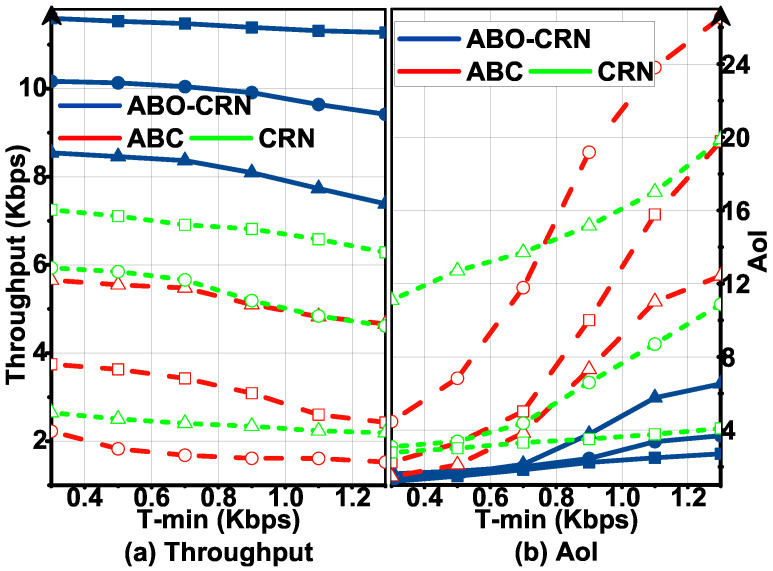
Throughput T and AoI versus $T_{min}$ under $P_{a}=$ 0.3 (circle), 0.6 (square), 0.9 (triangle) compared with that in the ABCs and CRNs, and $A_{max}=5$: (**a**) describes the throughput performance. (**b**) describes the AoI performance.

**Figure 9:**
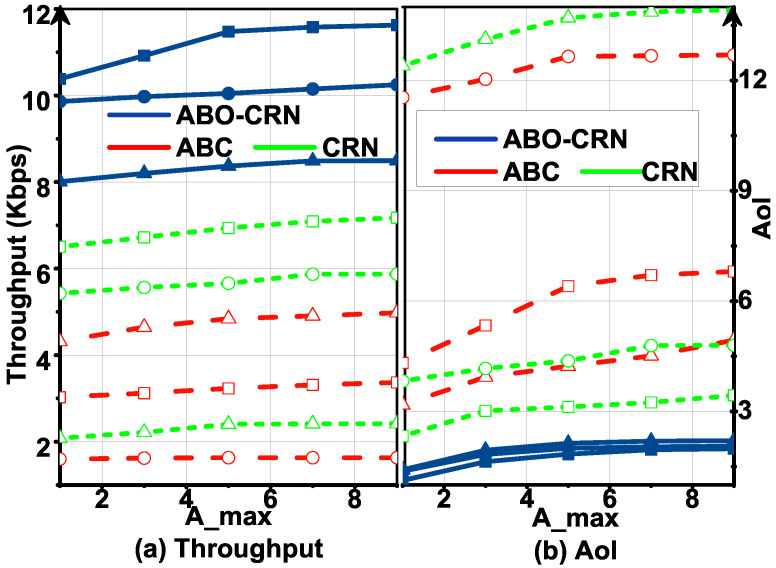
Throughput T and AoI versus $A_{max}$ under $P_{a}=$ 0.3 (circle), 0.6 (square), 0.9 (triangle) compared with that in the ABCs and CRNs, and $T_{min}=700$ bps: (**a**) describes the throughput performance. (**b**) describes the AoI performance.

Figure 8 plots T and AoI in the ABO-CRN, ABCs, and CRNs with $T_{min}$, and Figure 9 plots T and AoI in the ABO-CRN, ABCs, and CRNs with $A_{max}$, under $P_{a}=$ 0.3, 0.6, 0.9. It is obvious that T in the ABO-CRN is higher than that in the ABCs and CRNs, and AoI in the the ABO-CRN is lower than that in the ABCs and CRNs. When $P_{a}=$ 0.3 and 0.9, the AoI in the ABCs and CRNs are high, and when T is large, STs in the ABCs and CRNs can not satisfy the AoI constraint. The reason is explained as follows. When $P_{a}=$ 0.3, the channel keeps inactive for the majority part of the time. For the ABCs, AB mode transmission has a few opportunities to be executed. From Figure 6, we infer that, when $T_{min}$ is higher than the expected throughput achieved by each ST through AB mode transmission in a frame, STs in the ABCs are difficult to satisfy the AoI constraint by sacrificing the total throughput. Therefore, the throughput of the ABCs in this case keeps nearly unchanged. When $P_{a}=$ 0.9, the channel keeps active for the majority part of the time. For the CRNs, overlay mode transmission has few opportunities to be executed, hence the AoI of the CRNs is high, and the throughput of the CRNs keeps nearly unchanged. STs in the ABO-CRN execute AB mode transmission when the channel is active, and execute overlay mode transmission when the channel is inactive. As described in Equation (16), the expected throughput achieved by STs in the ABO-CRN is higher than that in the ABCs and CRNs. Therefore, the ABO-CRN achieves better throughput and AoI performances than that of the ABCs and CRNs.

## 6. Conclusions

We optimized the long-term throughput of the secondary network with the AoI constraint by jointly managing the time and energy for STs in the ABO-CRN, ABCs, and CRNs through DDPG. When the AoI constraint can not be satisfied, the impacts of time and energy allocation on the reward were investigated, and the corresponding reward functions was developed based on the channel states. We discussed the minimum throughput requirement and the maximum allowable AoI that are related to the throughput and AoI performances. We compared the throughput optimization scheme with the AoI constraint with T-O and A-O baseline schemes, and varied the minimum throughput requirement and maximum allowable AoI to evaluate the effects on the throughput and AoI performances of the secondary networks in the ABO-CRN, ABCs, and CRNs. We had following findings:

- Throughput of the ABO-CRN is close to the optimal throughput of T-O baseline scheme, and the AoI of the ABO-CRN is close to the optimal AoI of A-O baseline scheme. DDPG finds the optimal policy of time and energy management to optimize the throughput, and satisfies the AoI constraint at the same time.
- Throughput of the ABO-CRN is higher than that of A-O baseline scheme, and AoI of the ABO-CRN is lower than that of T-O baseline scheme. The observation validates the benefit of considering both throughput and AoI performances over only one metric.
- The ABO-CRN improves the throughput and AoI performances of the ABCs and CRNs. Even in extreme cases, such as the long time active channel state, the ABO-CRN obtains better throughput and AoI performances than the ABCs and CRNs.
- The lower bound of the maximum allowable AoI that makes STs satisfy the AoI constraint decreases with the total number of STs, and increases with the number of STs whose average throughput is smaller than the minimum throughput requirement.

## Figures and Tables

**Table 1:** Comparison Table of the Related works.

Scenario	Metric	Limitations
CRNs	Throughput [14,15], AoI [26,27,28]	Short-term optimization [14,15,27], single ST [14,15,26,27,28], single metric optimization, single resource management [14,15,26,27].
ABCs	Outage probability [19], backscatter efficiency [20], throughput [21], AoI [29]	Short-term optimization, single resource management [19,20,21], single metric optimization [19,20,21,29]
AB-CRNs	Throughput [22,23], coverage probability [24]	Short-term optimization [22,23], single ST [22,23,26], single metric optimization [22,23,24], single resource management [22,23].

**Table 2:** Parameter list.

Parameter	Description
*n*	The number of STs is *n* + 1
$s_{t}^{a}$	The channel state in frame *t*
$P_{a}$	The probability of the active channel state
*E*	The capacity of rechargeable capacitor
$e_{t,i}$	The allocated energy for overlay mode transmission of ST${}_{i}$
$\varepsilon_{t,i}$	The available energy of ST${}_{i}$ in frame *t*
$\alpha_{t,i}$	The duration of data transmission by ST${}_{i}$ in frame *t*
$T_{t}$	The total throughput of secondary network in frame *t*
$T_{t,i}$	The throughput of ST${}_{i}$ in frame *t*
$T_{t}^{A}$	The throughput of STs by AB mode transmission
$T_{t}^{O}$	The throughput of STs by overlay mode transmission
$T_{min}$	The minimum throughput requirement for each ST
*W*	The bandwidth
P	The transmit power of the PT
$g_{t,i}$	The channel gain from the PT to ST${}_{i}$ in frame *t*
$h_{t,i}$	The channel gain from ST${}_{i}$ to gateway in frame *t*
θ	The backscatter reflection coefficient
$\delta^{2}$	The variance of AWGN
$a_{t,i}$	The AoI of ST${}_{i}$ in frame *t*
$A_{max}$	The maximum allowable AoI

## Data Availability

Not applicable.

## Abbreviations

We used the abbreviations in this paper:

RF	Radio frequency
CR	Cognitive radio
CRN	CR network
AB	Ambient backscatter
ABC	AB communication
AB-CRN	AB-assisted CRN
ABO-CRN	AB-assisted overlay CRN
ABU-CRN	AB-assisted underlay CRN
DRL	Deep reinforcement learning
DDPG	Deep deterministic policy gradient
AoI	Age of information
PU	Primary user
PT	Primary transmitter
PR	Primary receiver
SU	Secondary user
ST	Secondary transmitter
SR	Secondary receiver
